# Career Choice and Attractiveness of Child and Adolescent Psychiatry as a Medical Specialty: A National French Questionnaire Survey

**DOI:** 10.3389/fpsyt.2021.560141

**Published:** 2021-02-18

**Authors:** Alexis Revet, Jean-Philippe Raynaud, Daniel Marcelli, Bruno Falissard, Nicole Catheline, Grégoire Benvegnu

**Affiliations:** ^1^Service Universitaire de Psychiatrie de l'Enfant et de l'Adolescent, CHU de Toulouse, Toulouse, France; ^2^UMR 1027, Inserm, Université Toulouse III, Toulouse, France; ^3^Past President of the Société Française de Psychiatrie de l'Enfant et de l'Adolescent et Disciplines Associées (SFPEADA), Clinique de Psychopathologie de l'Enfant et de l'Adolescent, Hôpital de la Salpêtrière, Paris, France; ^4^Université Paris-Saclay, UVSQ, Inserm, CESP, Villejuif, France; ^5^President of the Scientific Council of the Société Française de Psychiatrie de l'Enfant et de l'Adolescent et Disciplines Associées (SFPEADA), Clinique de Psychopathologie de l'Enfant et de l'Adolescent, Hôpital de la Salpêtrière, Paris, France

**Keywords:** child and adolescent psychiatry, education, medical students, career choice, France

## Abstract

**Context:** The shortage of child and adolescent psychiatrists in France affects access to early interventions and mental health services and impacts therapeutic practices and prescribing trends. This study aimed to describe factors associated with choosing child and adolescent psychiatry (CAP) as a career specialty and with assessing the level of attractiveness of this discipline and its predictors.

**Methods:** We generated a self-report questionnaire using a modified two-step Delphi approach. The survey was conducted from January 13 to February 16, 2020, and targeted French child and adolescent psychiatrists or psychiatrists, mainly working with children and/or adolescents. We used a logistic regression model to assess the factors associated with the perception of CAP as attractive. A thematic qualitative analysis of the free comments section of the questionnaire was performed.

**Results:** Of 863 doctors contacted by email, the response rate was 77.4% (668 respondents). Two-thirds of respondents were female and had an official specialization in CAP. One-third were aged between 31 and 40 years. The main reasons for choosing to specialize in CAP were interest in working with children (64.2%), interest in psychotherapy (52.8%), and influence of an internship in CAP during medical studies (46.0%), although only one-third of respondents actually did an internship. Over half of respondents (57.3%) identified personal factors as implicated in their choice of CAP, mainly personal psychotherapy (22.2%) and personal trauma (14.4%). Although only 58.4% of respondents perceived CAP as attractive, 97.8% had no regret about their choice, and 83.5% would make the same career choice today. A positive perception by respondents' surroundings for choosing CAP at the time of the choice was associated with a perception of CAP as currently attractive by respondents (odds ratio: 1.82; 95% confidence interval: 1.27–2.60; *p* = 0.001).

**Conclusion:** CAP is in crisis and faces many challenges in France, with an urgent need to redress its demographics. Many child and adolescent psychiatrists do not regret their choice and would choose the same specialty today. To increase its attractiveness, better visibility during medical school, enhanced academic recognition, and increased remuneration seem promising.

## Introduction

Child and adolescent psychiatry (CAP) is a relatively young medical specialty, which currently faces many challenges, as there is a tremendous increase in demand for child and adolescent mental health services but a major lack of CAP workforce to meet these demands (1–4). Epidemiological data now clearly show that CAP must be a public health priority in most countries in the world (5, 6). In France, psychiatry and CAP remain among the most stigmatized medical disciplines, and <6% of French medical students choose psychiatry as a specialty (7). The shortage of child and adolescent psychiatrists in France (8), which affects access to early interventions and mental health services and therapeutic practices and prescribing trends (9), has recently been highlighted (8, 10). It seems that postgraduate training in psychiatry and CAP has not grown at the same rate as other medical specialties, and these disciplines are still among the last to be chosen after the national competitive exam that concludes medical studies in France. The downward trend in CAP recruitment is not unique to France and has been recognized in many countries, including the United Kingdom (11), the United States (12), and Canada (13).

Reasons influencing the career choice of medical students in Western countries are complex (14, 15), ranging from socioeconomic factors (age, sex, marital status, and parents' socioeconomic status) to lifestyle considerations (working hours and level of income), and factors more directly related to the discipline (level of perceived-prestige, type of practice, and integrated clinical and research training during residency), or to the medical studies (internship during medical school and presence of a local mentor or role model) (16–19).

Child and adolescent psychiatrists need the competencies to deal with complex disorders that have their origins in the biological, psychological, systemic, contextual, and cultural domains (20). This level of complexity, as well as the lack of familiarity of medical students with this type of bio-psycho-social approach, may explain this apparent lack of attractiveness of CAP. However, it is essential to recognize that CAP is a medical discipline with a couple of specificities and many similarities with other medical fields. Within this perspective, it is important to explore specific factors associated with choosing CAP as a medical career.

Thus, the purpose of this study was 2-fold: to describe factors associated with choosing CAP as a medical specialty and with assessing the level of attractiveness of this discipline and its predictors among child and adolescent psychiatrists. The intent is to address the shortage of specialists in this field by suggesting solutions and guidance to improve CAP's visibility during medical school and its global attractiveness.

## Methods

### Questionnaire Development

We generated a self-report questionnaire using a modified two-step Delphi approach. The Delphi technique is a formal consensus method used in medical and health service research to obtain and integrate the views and opinions of experts in a particular field (21, 22). Using a multistage process, AR and GB first drew-up a list of 40 items related to factors influencing the choice of CAP as a medical specialty and to its attractiveness, based on a literature search (13, 23), and their own personal and professional experience. AR, GB, and JPR then ranked the item list by importance on a 10-point Likert scale, and these 40 items were then reduced and assembled in a 25-item questionnaire in the first round of the study. This first version of the questionnaire was then submitted to 26 members of the Scientific Committee of the French Society of Child and Adolescent Psychiatry and Allied Professions [*Société Française de Psychiatrie de l'Enfant et de l'Adolescent et Disciplines Associées* (SFPEADA)] who analyzed the questionnaire in-depth and proposed their corrections. A third round followed, during which experts were asked to revise their earlier answers in light of the corrections and feedbacks of other members of the Scientific Committee. A consensus emerged on this third version, and the final questionnaire included two sociodemographic items (age group and sex), three items regarding CAP education, three items focusing on the type of professional practice, nine items detailing the context and the reasons of choosing CAP as a medical specialty, and seven items exploring the respondent's current vision of CAP, especially in terms of attractiveness. The last section of the questionnaire consisted of free comments with no length limitation. An English version of the questionnaire is available from the corresponding author.

### Recipients and Estimation of the Number of Child and Adolescent Psychiatrists in France

To target all French child and adolescent psychiatrists or psychiatrists without official certification in CAP but mainly working with children and/or adolescents (frequent in France), we fastidiously used the following steps:

- To obtain mailing lists from national societies, such as the SFPEADA, and from the network of French professors in CAP;
- To identify French authors in the field of child and adolescent mental health by a systematic screening of the most important French journals in the field of CAP or psychoanalysis;
- To identify child and adolescent psychiatrists listed as experts in the highest court of justice in France (the Court of Cassation);
- Then, to identify through a systematic internet screening doctors working in each public departmental institution of CAP and to deduce their email addresses through a trial and error process (from the last name, first name, and internet domain of the institutions).

At the end of this search, we obtained 1,006 names of doctors working in a variety of clinic settings and 863 (85.8%) valid email addresses.

We then contacted the French Medical College to get precise and current data about the exact number of officially registered (i.e., officially certified) child and adolescent psychiatrists in France. According to these data, there are 425 certified and active child and adolescent psychiatrists and 189 certified ones who are officially retired but still active through private practice. Overall, 1,017 psychiatrists mainly worked with children and adolescents in France, among whom 440 are “active” retirees.

### Questionnaire Distribution

The survey was conducted from 13 January to 16 February 2020. To optimize the response rate, we used a repeat mailing approach according to a Cochrane-based approach (24). We re-mailed the questionnaire every 2 weeks to all doctors (two reminders in total). In accordance with French legal regulations, ethical approval was not required for this anonymous, questionnaire-based study.

### Statistical Analyses

Survey response data were analyzed using simple descriptive statistics. We then performed a logistic regression model to assess factors associated with the perception of CAP as attractive. Variables included were sex, age, the impact of personal factors on the choice of CAP, certification in CAP, main categories of patients seen in routine practice, reasons for choosing psychiatry as a medical specialty, perception of the choice of CAP as a specialty by respondents' surroundings, first job in CAP after graduation, and internship during medical school (in psychiatry, CAP or pediatrics). The significance level was set at 0.05. All statistical analyses were performed using RStudio software version 1.1.453 (The R Foundation for Statistical Computing, Vienna, Austria).

### Thematic Analysis

The qualitative analysis of the free comments section of the questionnaire was performed using the thematic analysis methodology, a qualitative descriptive approach for identifying, analyzing, and reporting patterns (themes) within data (25, 26). We followed several steps (27): first, we familiarized ourselves with data by transcribing, reading, and rereading them and noting down initial ideas; then, we generated initial codes by coding interesting features of the data systematically across the entire data set and collated codes into potential themes. The themes were then reviewed, meaning we checked if the themes worked in relation to the coded extracts and the entire data set, generating a thematic map; finally, we defined and named themes by refining the specifics of each theme and generating clear definitions and names for each theme. Presented in the results are a selection of vivid and compelling extract samples translated into English to illustrate the results of this analysis process.

## Results

### Number and Characteristics of Respondents

Of 863 doctors contacted by email, 668 responded, giving a 77.4% response rate. Among them, 442 indicated that they are qualified in CAP, of which 421 are still active, and 21 are retired. In theory, this means that our study covered 99.1% of certified and non-retired CAP in France (421 of 425).

Two-thirds of respondents were female (*n* = 459; 68.7%). Around one-third of respondents (*n* = 217; 32.5%) were aged between 31 and 40 years. Many respondents obtained their medical degree in psychiatry (*n* = 596; 89.2%), but 52 (7.8%) first graduated in family medicine and then changed to a CAP practice. Only 442 (66.2%) of these medical doctors mainly working in the field of child and adolescent mental health had an official specialization in CAP. A large portion of respondents (*n* = 275; 41.2%) worked in public non-academic institutions (i.e., regional hospitals), and 151 (22.6%) worked in a university hospital (i.e., an institution that combines the services of a hospital with the education of medical students and with medical research). However, 147 (22.0%) had a mixed practice, combining working in an academic or non-academic hospital, medico-social institutions, or private institutions. Thirty respondents (4.5%) were retired. Most respondents worked with preschoolers and schoolers (*n* = 195; 29.2%) and adolescents (*n* = 189; 28.3%). Detailed results are presented in Tables 1, 2.

**Table 1:** Sociodemographic and curriculum-related characteristics of respondents (*n* = 668).

	***n* (%)**
Sex	
Males	209 (31.3)
Females	459 (68.7)
Age	
25–30 years	27 (4.0)
31–35 years	116 (17.4)
36–40 years	101 (15.1)
41–45 years	77 (11.5)
46–50 years	70 (10.5)
51–55 years	72 (10.8)
56–60 years	88 (13.2)
61–65 years	64 (9.6)
>65 years	53 (7.9)
Discipline in which MD was obtained	
Psychiatry	596 (89.2)
Family medicine	52 (7.8)
Pediatrics	8 (1.2)
Neuropsychiatry	7 (1.0)
Other	5 (0.7)
Certification in child and adolescent psychiatry	
Yes	442 (66.2)
No	226 (33.8)
First job in child and adolescent psychiatry	
Yes	510 (76.3)
No	158 (23.7)

**Table 2:** Practice-related characteristics of respondents (*n* = 668).

	***n* (%)**
Main type of practice	
Public non-academic hospital	275 (41.2)
Public academic hospital^a^	151 (22.6)
Medico-social institution	41 (6.1)
Private institution (clinic or private office)	24 (3.6)
Retiree	30 (4.5)
Mix practice	147 (22.0)
Place of practice	
Medico-psychological center	386 (57.8)
Outpatient hospitalization	226 (33.8)
Specialized consultation	135 (20.2)
Part-time therapeutic center	128 (19.2)
Inpatient hospitalization	123 (18.4)
Medico-social institution	105 (15.7)
Emergency unit	86 (12.9)
Private office	83 (12.5)
Mobile team	69 (10.3)
Medico-psycho-pedagogical center	61 (9.1)
Adolescent center	57 (8.5)
Early action medico-social center	9 (1.3)
Other	102 (15.3)
Main categories of patients seen in routine practice	
Perinatal and infants	48 (7.2)
Toddlers (1–3 years old)	38 (5.7)
Preschoolers and schoolers (3–12 years old)	195 (29.2)
Adolescents (12–18 years old)	189 (28.3)
Young adults (16–25 years old)	17 (2.5)
Mix practice	181 (27.1)

a *An academic hospital or university hospital is an institution that combines the services of a hospital with the education of medical students and with medical research*.

### Factors for Choosing Child and Adolescent Psychiatry

Interest in psychiatry during medical school was the main reason for choosing this specialty as a career (*n* = 327; 49.0%), followed by vocation or long-held aspiration (*n* = 283; 42.4%). More than two-thirds of respondents (*n* = 478; 71.6%) did an internship in psychiatry during their medical studies, but only 223 (33.4%) did an internship in CAP. The main reasons for choosing to specialize in CAP were interest for work with children (64.2%), interest in psychotherapy (52.8%), and the influence of an internship in CAP during medical school (46.0%), whereas conditions of life and practice were the least advanced reasons to choose the specialty (7.2%). A narrow majority declared a positive perception by their surrounding for choosing CAP as a medical specialty (*n* = 367; 54.9%). Among the negative comments by respondents' surroundings regarding CAP, preconceived ideas on patients with mental illness were the most frequent (36.7%). A majority of respondents (*n* = 383; 57.3%) identified personal factors as implicated in their choice of CAP, of which personal psychotherapy (*n* = 148; 22.2%) and personal trauma (*n* = 96; 14.4%) were the most frequent (Table 3).

**Table 3:** Factors associated with the choice of child and adolescent psychiatry (*n* = 668).

	***n* (%)**
Reasons for choosing psychiatry as a medical specialty	
Interest in this specialty during medical school	327 (49.0)
Vocation (long-held aspiration)	283 (42.4)
Change of orientation (another medical specialty initiallychosen)	34 (5.1)
Default choice	24 (3.6)
Internship in psychiatry during medical school	
Yes	478 (71.6)
No	190 (28.4)
Internship in CAP during medical school	
Yes	223 (33.4)
No	445 (66.6)
Internship in pediatrics during medical school	
Yes	556 (83.2)
No	112 (16.8)
Reasons for choosing to specialize in CAP	
Interest in the work with children	429 (64.2)
Interest for psychotherapy	353 (52.8)
Influence of an internship in child and adolescent psychiatry during medical studies	307 (46.0)
Interest in the work with adolescents	297 (44.5)
Developmental approach in child and adolescent psychiatry	286 (42.8)
Psychanalytic approach in child and adolescent psychiatry	271 (40.6)
Local attractiveness of the discipline	122 (18.3)
Refusal of adult psychiatry	94 (14.1)
Conditions of life and of practice	48 (7.2)
Other	63 (9.4)
Perception of the choice of CAP as a specialty by surroundings	
Positive perception	367 (54.9)
Neutral perception	227 (33)
Negative perception	74 (11.1)
Type of negative comments concerning CAP by surroundings	
Received ideas on patients suffering from mentalillness	245 (36.7)
No direct comment of the surroundings	205 (30.7)
Fear to be exposed to mental suffering	199 (29.8)
Abandonment of somatic/organic medicine	194 (29.0)
Lack of prestige of the discipline	153 (22.9)
Low salary	136 (20.4)
Other	30 (4.5)
Impact of personal factors on the choice of CAP	
Yes	383 (57.3)
No	285 (42.7)
Personal factors with an impact on the choice of CAP	
Personal psychotherapy	148 (22.2)
Personal trauma	96 (14.4)
Mental illness in the surroundings	91 (13.6)
Psychiatrist or child and adolescent psychiatrist in the surroundings	74 (11.1)
Other	112 (16.8)

*CAP, child and adolescent psychiatry*.

### Attractiveness of Child and Adolescent Psychiatry Specialty

Although a relatively narrow majority of respondents (*n* = 390; 58.4%) perceived CAP as an attractive medical specialty, an overwhelming majority had no regret about their choice (*n* = 653; 97.8%), would recommend it to medical students (*n* = 599; 89.7%), and would choose it again today (*n* = 558; 83.5%). The main reasons of attractiveness were diversity of clinical practice (*n* = 382; 57.2%), multidisciplinary approach (*n* = 373; 55.8%), and strong connections with other medical specialties (*n* = 269; 40.3%). CAP's lack of attractiveness was mainly attributed to a lack of resources (*n* = 356; 53.3), lack of recognition of the discipline (*n* = 234; 35.0%), and the difficulty of clinical and social situations encountered in routine practice (*n* = 232; 34.7%). Lastly, the main factors that could increase its attractiveness were better visibility during medical school (*n* = 533; 27.9%), better pay of child and adolescent psychiatrists (*n* = 410; 21.5%), and better academic recognition (*n* = 383; 20.1%) (Table 4).

**Table 4:** Perceived attractiveness of child and adolescent psychiatry (*n* = 668).

	***n* (%)**
Perceptiveness of attractiveness of CAP	
Attractive	390 (58.4)
Not attractive	278 (41.6)
Reasons for attractiveness	
Diversity of the clinical practice	382 (57.2)
Multidisciplinary approach	373 (55.8)
Strong connections with other medical specialties (pediatrics, genetics…)	269 (40.3)
Practice compatible with a family life	121 (18.1)
Professional opportunities	96 (14.4)
Other	42 (6.3)
Reasons for non-attractiveness	
Lack of resources (inpatients and outpatients' units)	356 (53.3)
Lack of recognition of the discipline	234 (35.0)
Difficulties of clinical and social situations encountered in routine practice	232 (34.7)
Low level of consideration of the discipline by other medical specialties	202 (30.2)
Lower salary than in other specialties	191 (28.6)
Violence of psychiatric field in general	53 (7.9)
Other	60 (9.0)
Factors that could increase the attractiveness of CAP	
Better visibility in general of the discipline during medical school	533 (27.9)
Better level of salary of child and adolescent psychiatrists	410 (21.5)
Better academic recognition of the discipline	383 (20.1)
Development and integration of research activities to the training	247 (12.9)
Separation and individualization of child and adolescent psychiatry from adult psychiatry with distinct training and residency	160 (8.4)
Increase in the length of the residency	54 (2.8)
Other	122 (6.4)
Recommendation of CAP as a good choice of medical specialty to students in medicine	
Yes	599 (89.7)
No	69 (10.3)
Regrets concerning the choice of CAP as a medical specialty	
Yes	15 (2.2)
No	653 (97.8)
Choice of medical specialty which would be done today	
CAP	558 (83.5)
Psychiatry	19 (2.8)
Pediatrics	12 (1.8)
Family medicine	7 (1)
Other	72 (10.8)

*CAP, child and adolescent psychiatry*.

In multivariate analysis, the only factor associated with a perception of CAP as currently attractive was a positive perception by respondents' surroundings (compared with a neutral perception) for choosing CAP as a medical specialty at the time of the choice [odds ratio (OR): 1.82; 95% confidence interval (CI): 1.27–2.60; *p* = 0.001] (Table 5).

**Table 5:** Multivariate model assessing the relationship between the perception of child and adolescent psychiatry as attractive and the detailed effects of different covariates.

**Variables**	**OR (95 CI)**	***p***
Sex		
Male	1.02 (0.70–1.47)	0.9
Age	1.04 (1.02–1.05)	<0.00001
Impact of personal factors on the choice of CAP	1.00 (0.72–1.38)	1
Certification in CAP	0.90 (0.63–1.29)	0.6
Main categories of patients seen in routine practice		
Perinatal and infants	1.42 (0.71–2.83)	0.3
Toddlers	1.66 (0.76–3.60)	0.2
Preschoolers and schoolers	0.69 (0.45–1.05)	0.08
Adolescents	1	-
Young adults	0.79 (0.28–2.23)	0.7
Mix practice	1.17 (0.75–1.81)	0.5
Reasons for choosing psychiatry as a medical specialty		
Interest in this specialty during medical school	1	-
Vocation (long-held aspiration)	0.94 (0.66–1.33)	0.7
Change of orientation	0.77 (0.36–1.63)	0.5
Default choice	1.63 (0.65–4.06)	0.3
Perception of the choice of CAP as a specialty by surroundings		
Negative perception	1.10 (0.64–1.90)	0.7
Neutral perception	1	-
Positive perception	1.82 (1.27–2.60)	0.001
First job in CAP after graduation	0.90 (0.59–1.40)	0.7
Internship in psychiatry during medical school	1.01 (0.70–1.45)	1
Internship in CAP during medical school	1.25 (0.87–1.79)	0.2
Internship in pediatrics during medical school	1.18 (0.76–1.85)	0.5

*CAP, child and adolescent psychiatry*.

### Personal Comments of the Respondents

Of 668 doctors who participated in this study, 243 (36.4%) wrote a free comment at the end of the questionnaire. The proportion of respondents who wrote a free comment increased with age, from 26% in the 25–30 years old group to 53% in the more than 65 years old group. Comments could be classified into three main categories: propositions to increase the attractiveness of CAP, negative comments on the current situation, and positive comments on the intrinsic attractiveness of CAP (Table 6). No clear relation appeared between the age of the respondents and the nature of comments.

**Table 6:** Themes extracted from the thematic analysis and extracts from personal comments of the respondents (*n* = 243).

**Themes**	**Number of comments *n* (%)**	**Extracts**
Proposition to increase attractiveness of CAP	137 (56.4)	
Propositions to revitalize and promote CAP	69 (28.4)	
Better pay	20 (8.2)	“In private practice, your salary only is not sufficient to live.” “Consultations should be better paid because they imply a lot of different interventions: liaison activity, certificates' writing, work with family, etc.”
Better image (at political, social, and medical levels)	19 (7.8)	“Stronger political and financial support is required, as well as a better media exposure of all fields related to childhood (CAP, pediatrics, child protection, juvenile justice, handicap, education, etc.).” “Overall, psychiatry appears strange and mysterious to other medical specialties and even more to patients and their families. It is important to be more transparent and clearer in our practice, because it would reassure, appease conflicts and therefore lead to a better recognition of our work.”
Separation between CAP and adult psychiatry	10 (4.1)	“CAP must strengthen, increase its legitimacy and credibility, which will be possible only through an individualization process. CAP should keep strong connections with adult psychiatry but should also be in position to be able to speak for itself and to be directly represented.” “It is essential to separate CAP from adult psychiatry because current training in CAP is drown in adult ones et does not prepare to this very specific profession, which actually has very little in common with adult psychiatry.”
Propositions to improve training in CAP	68 (28.0)	
Lack of training	37 (15.2)	“Teaching of CAP during medical studies is very limited. Moreover, the recent reform of medical studies reduced opportunities to perform an internship in a CAP department.”
Theoretical divide	9 (3.7)	“The lack of respect of the different field and approaches in psychiatry, by other or “concurrent” fields, hinder CAP's attractivity.” “A greater coherence in our practices and theoretical frameworks is warranted to promote and clarify our identity.”
Critics of psychoanalysis	13 (5.3)	“It is more than urgent to clearly separate psychoanalysis from psychiatry, and more specifically from CAP, in order to develop valid and evidence-based approaches. It is an ethical imperative!” “In France, our specialty has been devastated by Freudian or Lacanian theories, during the seventies and the eighties. The lack of consistence and depth of the psychopathology exclusively derived from psychoanalysis has been disastrous for French CAP.” “We should put psychanalysis back at the center of CAP's practice, but in collaboration and under the light of neurosciences and other scientific recent data. It is an important practical and theoretical tool, and psychologists, who are not trained enough in semiology and psychopathology, should not have the monopoly of it.” “Psychoanalytical theory, of course within an integrative perspective, should continue to be taught to residents in CAP.”
Negative comments on the current situation	84 (34.6)	
Lack of resources affecting attractiveness of CAP	45 (18.5)	“There is an inescapable and growing gap between patients' needs and the means at our disposal to fulfill them. It is more and more unbearable to impose an indecent waiting list to the psychological suffering of our patients and their families. Our young colleagues in training are clearly aware of this situation and it is one of the main reasons which shift them away from CAP.”
Personal weariness	39 (16.0)	“For young child and adolescent psychiatrists, there is a real disillusionment with respect to their current conditions of practice. In the long run, it raises the question of whether they should continue their job or not… They have a feeling of being constantly solicited in their practice, while their commitment and their job is not recognized.” “I have grieved CAP and I only hope to hold on for over four years, but I am not sure of being capable of that… The “patient child and adolescent psychiatry” is dying.”
Positive comments on the intrinsic attractiveness of CAP	37 (15.2)	“I find my job exciting, I keep training, learning new things, meeting enthusiast colleagues, while colleagues from other medical fields are stuck in a routine and get bored.” “CAP is probably the most comprehensive and the richest medical specialty.”

*CAP, child and adolescent psychiatry*.

## Discussion

### Key Findings

To our knowledge, this study is the first to attempt to entirely cover certified and non-retired CAP in France. Around two-thirds of respondents were female and had an official specialization in CAP, and one-third were aged between 31 and 40 years. The main reasons for choosing to specialize in CAP were interest in working with children, interest in psychotherapy, and influence of an internship in CAP during medical studies (although only one-third of respondents actually did an internship). Most respondents identified personal factors as implicated in their choice of CAP, mainly personal psychotherapy and personal trauma. Although a relatively narrow majority of respondents perceived CAP as attractive, an overwhelming majority had no regrets about their choice and would choose it again today. Interestingly, a positive perception by respondents' surroundings for choosing CAP as a medical specialty at the time of the choice was associated with a perception of CAP as currently attractive. The main factors that could increase its attractiveness were better visibility during medical school, better pay, and better academic recognition.

### Discussion of Research Findings

Cohen et al. (2) pointed out that French CAP is in crisis, with the number of child and adolescent psychiatrists halving during the last decade and a rise in territorial inequalities, whereas the need for CAP activity increased drastically during the same period as well as global social demand. This situation led to a prolonged time to access treatment, which can sometimes exceed 1.5 years. The strict *numerus clausus*, which was applied until recently in France, consists of a competitive exam at the end of the first year of medical studies, limiting the number of medical students; furthermore, another exam in the later years of medical studies is required for choosing medical specialties. We can speculate that this has had a major influence on the number of medical students completing their studies and eventually choosing the specialty. This recruitment crisis is not specific to CAP and also concerns adult psychiatry in France as in many countries (28–31). This lack of CAP specialists logically explains the fact that a third of respondents did not have official certification in CAP. Although tasks shifting to non-specialist providers has been proposed to reduce the treatment gap for children and adolescents, and adults with mental disorders (32), increasing the involvement of non-specialist providers may also be a problem with regard to the complexity of the CAP specialty, which combines unification theory, developmental approaches, precise and subtle clinical practice, and psychopathology, ranging from phenomenology to psychoanalysis, but also neurosciences and social sciences. As stated by Falissard in a recent editorial (3): “Well-designed flowcharts can indeed be useful to help nurses or general practitioners to deal with young patients in countries where child and adolescent psychiatrists are lacking. Putting symptoms into the context of a family, a culture and a patient's specific development requires much more than that, and this is the day-to-day activity of a well-trained physician.” Of course, continuing medical education courses are useful, but they are mainly designed to maintain competence and teach new and developing areas of the medical specialty, once chosen.

This situation is not limited to France. Overall, approximately half of all mental disorders emerge before 14 years of age and three-quarters by 25 years (5, 33). Moreover, 25% of disability-adjusted life years for mental and substance use disorders occur in youth (34). Despite increasing recognition of the importance of mental health prevention and promotion in children and adolescents, there is still a dramatic gap between needs and resource availability (35). Worldwide, the very low number of child and adolescent psychiatrists is particularly worrying, and it is even more marked in low-income countries. Although in high-income countries, this number is 1.19 per 100.000 youth, in low-and middle-income countries, it is <0.1 per 100.000 population (36). Furthermore, there is evidence for the increasing prevalence of some child and adolescent mental disorders, in particular, depression (37), substance use disorders (38), and autism spectrum disorders (39), without proportionate expansion in the youth mental health workforce and services. This situation certainly contributes to a kind of vicious circle. The fact that societies are not investing enough in the psychiatric care of children and adolescents leads to long waiting lists and overwhelmed services, which in turn comes with difficulty to recruit young doctors, who are not interested or even afraid of implicating themselves into services that lack basic funding. In this context, conclusions from a working group including experts from the International Association for Child and Adolescent Psychiatry and Allied Professions (IACAPAP), the World Association for Infant Mental Health, the Section on Child and Adolescent Psychiatry of the World Psychiatric Association, the International Society for Adolescent Psychiatry and Psychology, the UN Special Rapporteur on the Right to Health, and representatives of the World Health Organization Department of Mental Health and Substance Abuse were published in a recent editorial (4). They outlined four consensus priorities for CAP over the next decade: increase the workforce necessary for providing care for children, adolescents, and families facing mental disorders; reorientate child and adolescent mental health services to be more responsive to broader public health needs; increase research and research training while also integrating new research findings promptly and efficiently into clinical practice and research training; and increase efforts in advocacy.

One of the most interesting results of this study lies in an apparent paradox. Indeed, although a relatively narrow majority of respondents perceived CAP as attractive, an overwhelming majority had no regrets about their choice, would choose it again today, and would even recommend this choice to medical students. Moreover, the most reported reasons of CAP's choice (affinity toward children, interest for psychotherapy, early internship, and personal factors) and attractiveness (diversity of clinical practice and multidisciplinary approach) clearly illustrate the fact that this specialty seems to be chosen and exercised with passion, the conditions of life and practice being the least advanced reasons to choose CAP. An interest in psychotherapy, both as a professional practice and a personal experience, contrasts with the lack of official training provided during residency and the high level of heterogeneity in personal training offering, which depends heavily on the region and the university hospitals throughout France. This is especially problematic with regard to the diversity of psychotherapy approaches used in CAP, from cognitive–behavioral therapy to psychanalysis, and the time required to acquire skills and experience (20). Interestingly, an online survey (40) conducted in psychiatric trainees from 22 countries found that the risk of severe burnout was higher in psychiatric trainees who had not opted for psychiatry as a first career choice. In view of this, the association between a positive perception by respondents' surroundings for choosing CAP as a medical specialty at the time of the choice and a perception of CAP as currently attractive may be a clear sign of the overall need for recognition of French child and adolescent psychiatrists, either at a global level (better visibility during medical studies and better academic recognition) or a personal level (better pay).

The fact that early exposure seems to be one of the most important aspects of recruitment is in line with results from previous studies focusing on psychiatry (41, 42) and other medical fields (19, 43). It is indeed important to ensure that CAP is sufficiently represented in undergraduate medical education, both in teaching hours and internships. Advocacy of its key role in the overall health-care system will be a determinant to ensure respect for CAP and other medical specialties. The importance of role models, supervisors, professors, and senior residents has been highlighted in psychiatry (44, 45), as in other specialties (46). Although recent efforts to support better academic recognition of CAP have been made (8), CAP still lags behind other medical specialties with a little more than 40 university professors and associate professors (by way of comparison, there are around 100 in adult psychiatry and around 200 in pediatrics). In addition, there is a French deficit of research in psychiatry, with only 2% of the public funding for medical research allocated to psychiatry research compared with 7% in the United Kingdom and 16% in the United States (47).

CAP's foundation in France only dates back to 1937, when Professor Georges Heuyer organized the first World Congress of Child Psychiatry in Paris, which offered an official recognition of the academic dimension of this new discipline. Although CAP has found its roots in a combination of medicine (adult psychiatry and pediatrics), psychology, and exchanges with various professionals involved with child and adolescent care and education, it is still a subspecialty of adult psychiatry, which means that French child and adolescent psychiatrists first have to specialize in psychiatry before having the possibility to specialize in CAP. In this regard, another key point will certainly be its progressive individualization from adult psychiatry, as is the case in Germany, Austria, or Switzerland, for instance. This will reinforce its visibility and perception as an autonomous medical specialty and not only a subspecialty or an option.

Recent European and international initiatives have been developed aimed to improve CAP's attractiveness, training facilities, and professional opportunities for trainees and early career child and adolescent psychiatrists. In line with the results from two studies (48, 49) conducted in European CAP trainees, which have highlighted the need for an agreed national curriculum in all European countries, the European Union of Medical Specialists Section for Child and Adolescent Psychiatry has proposed a framework that could serve as a template for further development (50). Online training support, such as the IACAPAP textbook (freely accessible here: https://iacapap.org/iacapap-textbook-of-child-and-adolescent-mental-health/), the IACAPAP Massive Open Online Course “Essentials of Child and Adolescent Psychiatry across the World” (freely accessible here: https://iacapap.org/essentials-of-child-and-adolescent-psychiatry-across-the-world/), or the European Psychiatric Association online course “Introduction to CBT” (accessible here: https://www.europsy.net/epa-online-course-on-cbt/), also offers valuable resources (51). Lastly, the European Society for Child and Adolescent Psychiatry (ESCAP) Research Academy, a network of young international researchers, which promotes research excellence and collaborative work among young clinician–scientists in the field of CAP (52), could also contribute to give a strong impact on the future of research in CAP (53, 54).

### Strengths and Limitations of the Study

The main strength of this study is a large number of participants and the high return rate (77.4%), which increases the validity of this cross-sectional study. Moreover, the fact that, in theory, this study covered 99.1% of certified and non-retired CAP in France means these data offer accurate sociodemographic information about French child and adolescent psychiatrists. Another strength lies in the multistage validation process of the questionnaire, which includes the participation of the 26 members of the Scientific Committee of the French Society of Child and Adolescent Psychiatry and Allied Professions.

However, this study also has some limitations. First, our results are influenced by both national and local health system peculiarities and cannot be directly transposed to other countries, which might be especially true in France, where adult psychiatry and moreover CAP organization and practices present some strong specificities (55, 56). Second, the tendency of social expectancy is a systematic bias of questionnaire surveys. It may be due to social desirability bias, which is explained by individuals responding in a way that they think is socially acceptable, for instance, causing them to over-report their satisfaction levels (57), or to courtesy, related to social desirability and implying that respondents have a tendency to give answers that they believe the interviewer wants to hear, rather than what they really feel (58). We tried to reduce these biases using an anonymous approach. Finally, our results obviously reflect the perceived and, therefore, possibly biased factors of choice of respondents and could differ from more objective sociodemographic data.

## Conclusion

The high response rate to this questionnaire study may be an indicator of the will and the need of French child and adolescent psychiatrists to be heard, both in their engagement and their love of their profession and in their feeling of exhaustion and conviction that a strong political and academic support must be provided to an endangered but fascinating medical specialty.

## Data Availability Statement

The dataset and the R script supporting the findings of this study are available from the corresponding author, Alexis Revet, upon reasonable request.

## Ethics Statement

Ethics committee approval was not required for this anonymous web-based questionnaire study.

## Author Contributions

AR, GB, and J-PR designed the first version of the questionnaire, which was then corrected and improved by NC, DM, and BF. AR and GB designed the study and wrote the protocol. AR oversaw all aspects of the research, conducted the analyses and produced the first draft of the manuscript. GB drafted portions and substantively edited all drafts of the manuscript. AR, GB, and J-PR analyzed the data. AR, GB, J-PR, NC, DM, and BF contributed to the interpretation of the data. J-PR, NC, DM, and BF critically revised the manuscript for important intellectual content. AR takes responsibility for the integrity of the data and the accuracy of the data analysis. All authors contributed to and have approved the final manuscript.

## Conflict of Interest

The authors declare that the research was conducted in the absence of any commercial or financial relationships that could be construed as a potential conflict of interest.

## References

[1] Sargeant JK Adey T McGregor F Pearce P Quinn D Milev R. Psychiatric human resources planning in Canada. Position paper. Canadian psychiatric association. Can J Psychiatry. (2010) 55:1–20. 20860107

[2] Cohen D Baubet T Duverger P Nezelof S Raynaud JP Rolland AC. Quel futur pour la psychiatrie de l'enfant et de l'adolescent ? Neuropsychiatr Enfance Adolesc. (2018) 66:191–3. 10.1016/j.neurenf.2018.05.003

[3] Falissard B. Thinking the future of child and adolescent psychiatry: what are we talking about? Eur Child Adolesc Psychiatry. (2018) 27:1519–21. 10.1007/s00787-018-1252-7 30406845

[4] Skokauskas N Fung D Flaherty LT von Klitzing K Puras D Servili C. Shaping the future of child and adolescent psychiatry. Child Adolesc Psychiatry Ment Health. (2019) 13:19. 10.1186/s13034-019-0279-y 31007713 PMC6458731

[5] Kessler RC Amminger GP Aguilar-Gaxiola S Alonso J Lee S Ustün TB. Age of onset of mental disorders: a review of recent literature. Curr Opin Psychiatry. (2007) 20:359–64. 10.1097/YCO.0b013e32816ebc8c 17551351 PMC1925038

[6] Gore FM Bloem PJN Patton GC Ferguson J Joseph V Coffey C. Global burden of disease in young people aged 10-24 years: a systematic analysis. Lancet Lond Engl. (2011) 377:2093–102. 10.1016/S0140-6736(11)60512-6 21652063

[7] Andlauer O Van Effenterre A Haffen E Sechter D Farooq K Lydall G. Encouraging French medical students to choose a career in psychiatry: how and why? Int Rev Psychiatry Abingdon Engl. (2013) 25:460–5. 10.3109/09540261.2013.821404 24032502

[8] Milon A Amiel M. Rapport d'information du Sénat Fait au Nom de la Mission d'information sur la Situation de la Psychiatrie Des Mineurs en France. (2017). Available online at: https://www.senat.fr/notice-rapport/2016/r16-494-notice.html (accessed March 4, 2020).

[9] Revet A Montastruc F Raynaud JP Baricault B Montastruc JL Lapeyre-Mestre M. Trends and patterns of antidepressant use in French children and adolescents from 2009 to 2016: a population-based study in the French health insurance database. J Clin Psychopharmacol. (2018) 38:327–35. 10.1097/JCP.0000000000000891 29851707

[10] Lopez A Turan-Pelletier G. Organisation et fonctionnement du dispositif de soins psychiatriques, 60 ans après la circulaire du 15 mars 1960. Rapport IGAS (2017). Available online at: http://www.igas.gouv.fr/IMG/pdf/2017-064R-Tome_I_rapport.pdf (accessed March 4, 2020).

[11] Brockington I Mumford D. Recruitment into psychiatry. Br J Psychiatry J Ment Sci. (2002) 180:307–12. 10.1192/bjp.180.4.307 11925352

[12] Vernon DJ Salsberg E Erikson C Kirch DG. Planning the future mental health workforce: with progress on coverage, what role will psychiatrists play? Acad Psychiatry. (2009) 33:187–92. 10.1176/appi.ap.33.3.187 19574512

[13] Volpe T Boydell KM Pignatiello A. Choosing child and adolescent psychiatry: factors influencing medical students. J Can Acad Child Adolesc Psychiatry. (2013) 22:260–7. 24223044 PMC3825465

[14] Wright B Scott I Woloschuk W Brenneis F Bradley J. Career choice of new medical students at three Canadian universities: family medicine versus specialty medicine. CMAJ. (2004) 170:1920–4. 10.1503/cmaj.1031111 15210640 PMC421719

[15] Puertas EB Arósquipa C Gutiérrez D. Factors that influence a career choice in primary care among medical students from high-, middle-, and low-income countries: a systematic review. Rev Panam Salud Publica Pan Am J Public Health. (2013) 34:351–8. 24553763

[16] Goldacre MJ Laxton L Harrison EM Richards JMJ Lambert TW Parks RW. Early career choices and successful career progression in surgery in the UK: prospective cohort studies. BMC Surg. (2010) 10:32. 10.1186/1471-2482-10-32 21044317 PMC2987756

[17] Lefevre JH Roupret M Kerneis S Karila L. Career choices of medical students: a national survey of 1780 students. Med Educ. (2010) 44:603–12. 10.1111/j.1365-2923.2010.03707.x 20604857

[18] Kiolbassa K Miksch A Hermann K Loh A Szecsenyi J Joos S. Becoming a general practitioner–which factors have most impact on career choice of medical students? BMC Fam Pract. (2011) 12:25. 10.1186/1471-2296-12-25 21549017 PMC3112095

[19] Kawamoto R Ninomiya D Kasai Y Kusunoki T Ohtsuka N Kumagi T. Factors associated with the choice of general medicine as a career among Japanese medical students. Med Educ Online. (2016) 21:29448. 10.3402/meo.v21.29448 27172894 PMC4865794

[20] Deschamps P Hebebrand J Jacobs B Robertson P Anagnostopoulos DC Banaschewski T. Training for child and adolescent psychiatry in the twenty-first century. Eur Child Adolesc Psychiatry. (2020) 29:3–9. 10.1007/s00787-019-01467-6 31950371 PMC6987048

[21] Jones J Hunter D. Consensus methods for medical and health services research. BMJ. (1995) 311:376–80. 10.1136/bmj.311.7001.376 7640549 PMC2550437

[22] Powell C. The Delphi technique: myths and realities. J Adv Nurs. (2003) 41:376–82. 10.1046/j.1365-2648.2003.02537.x 12581103

[23] Süß M Benson S Herbstreit S Dudda M Knobe M Hebebrand J. [Where did all the men in child and adolescent psychiatry and psychotherapy go? The influence of gender on the choice of specialization]. Z Kinder Jugendpsychiatr Psychother. (2019) 48:194–203. 10.1024/1422-4917/a000692 31657662

[24] Edwards PJ Roberts I Clarke MJ Diguiseppi C Wentz R Kwan I. Methods to increase response to postal and electronic questionnaires. Cochrane Database Syst Rev. (2009) 3:MR000008. 10.1002/14651858.MR000008.pub4 19588449 PMC8941848

[25] Vaismoradi M Turunen H Bondas T. Content analysis and thematic analysis: implications for conducting a qualitative descriptive study. Nurs Health Sci. (2013) 15:398–405. 10.1111/nhs.12048 23480423

[26] Castleberry A Nolen A. Thematic analysis of qualitative research data: is it as easy as it sounds? Curr Pharm Teach Learn. (2018) 10:807–15. 10.1016/j.cptl.2018.03.019 30025784

[27] Braun V Clarke V. Using thematic analysis in psychology. Qual Res Psychol. (2006) 3:77–101. 10.1191/1478088706qp063oa

[28] Saxena S Thornicroft G Knapp M Whiteford H. Resources for mental health: scarcity, inequity, and inefficiency. Lancet Lond Engl. (2007) 370:878–89. 10.1016/S0140-6736(07)61239-2 17804062

[29] Oyebode F Humphreys M. The future of psychiatry. Br J Psychiatry J Ment Sci. (2011) 199:439–40. 10.1192/bjp.bp.111.092338 22130743

[30] Katschnig H. Are psychiatrists an endangered species? Observations on internal and external challenges to the profession. World Psychiatry Off J World Psychiatr Assoc WPA. (2010) 9:21–8. 10.1002/j.2051-5545.2010.tb00257.x 20148149 PMC2816922

[31] Barkil-Oteo A. Psychiatry's identity crisis. Lancet Lond Engl. (2012) 379:2428. 10.1016/S0140-6736(12)61067-8 22748589

[32] Patel V Maj M Flisher AJ De Silva MJ Koschorke M Prince M. Reducing the treatment gap for mental disorders: a WPA survey. World Psychiatry Off J World Psychiatr Assoc WPA. (2010) 9:169–76. 10.1002/j.2051-5545.2010.tb00305.x 20975864 PMC2953637

[33] Kessler RC Berglund P Demler O Jin R Merikangas KR Walters EE. Lifetime prevalence and age-of-onset distributions of DSM-IV disorders in the national comorbidity survey replication. Arch Gen Psychiatry. (2005) 62:593–602. 10.1001/archpsyc.62.6.593 15939837

[34] Lopez AD Mathers CD Ezzati M Jamison DT Murray CJ. Global Burden of Disease and Risk Factors. Washington, DC: World Bank (2006). 10.1596/978-0-8213-6262-4 21250374

[35] Kieling C Baker-Henningham H Belfer M Conti G Ertem I Omigbodun O. Child and adolescent mental health worldwide: evidence for action. Lancet Lond Engl. (2011) 378:1515–25. 10.1016/S0140-6736(11)60827-1 22008427

[36] WHO. Mental Health ATLAS. WHO (2017). Available online at: http://www.who.int/mental_health/evidence/atlas/mental_health_atlas_2017/en/ (accessed January 6, 2021).

[37] Mojtabai R Olfson M Han B. National trends in the prevalence and treatment of depression in adolescents and young adults. Pediatrics. (2016) 138:e20161878. 10.1542/peds.2016-1878 27940701 PMC5127071

[38] Merikangas KR McClair VL. Epidemiology of substance use disorders. Hum Genet. (2012) 131:779–89. 10.1007/s00439-012-1168-0 22543841 PMC4408274

[39] Matson JL Kozlowski AM. The increasing prevalence of autism spectrum disorders. Res Autism Spectr Disord. (2011) 5:418–25. 10.1016/j.rasd.2010.06.00426048041

[40] Jovanović N Podlesek A Volpe U Barrett E Ferrari S Rojnic Kuzman M. Burnout syndrome among psychiatric trainees in 22 countries: risk increased by long working hours, lack of supervision, and psychiatry not being first career choice. Eur Psychiatry J Assoc Eur Psychiatr. (2016) 32:34–41. 10.1016/j.eurpsy.2015.10.007 26802982

[41] Galeazzi GM Secchi C Curci P. Current factors affecting the choice of psychiatry as a specialty: an Italian study. Acad Psychiatry. (2003) 27:74–81. 10.1176/appi.ap.27.2.74 12824106

[42] Denman M Oyebode F Greening J. Reasons for choosing to specialise in psychiatry: differences between core psychiatry trainees and consultant psychiatrists. BJPsych Bull. (2016) 40:19–23. 10.1192/pb.bp.114.048678 26958354 PMC4768842

[43] Harris MG Gavel PH Young JR. Factors influencing the choice of specialty of Australian medical graduates. Med J Aust. (2005) 183:295–300. 10.5694/j.1326-5377.2005.tb07058.x 16167868

[44] Manassis K Katz M Lofchy J Wiesenthal S. Choosing a career in psychiatry: influential factors within a medical school program. Acad Psychiatry. (2006) 30:325–9. 10.1176/appi.ap.30.4.325 16908613

[45] Tamaskar P McGinnis RA. Declining student interest in psychiatry. JAMA. (2002) 287:1859. 10.1001/jama.287.14.1859-JMS0410-5-1 11939877

[46] Jordan J Brown JB Russell G. Choosing family medicine. What influences medical students? Can Fam Physician Med Fam Can. (2003) 49:1131–7. 14526865 PMC2214282

[47] Chevreul K McDaid D Farmer CM Prigent A Park AL Leboyer M. Public and nonprofit funding for research on mental disorders in France, the United Kingdom, and the United States. J Clin Psychiatry. (2012) 73:e906–12. 10.4088/JCP.11r07418 22901361

[48] Simmons M Barrett E Wilkinson P Pacherova L. Trainee experiences of child and adolescent psychiatry (CAP) training in Europe: 2010-2011 survey of the European federation of psychiatric trainees (EFPT) CAP working group. Eur Child Adolesc Psychiatry. (2012) 21:433–42. 10.1007/s00787-012-0275-8 22618017

[49] Barrett E Jacobs B Klasen H Herguner S Agnafors S Banjac V. The child and adolescent psychiatry: study of training in Europe (CAP-STATE). Eur Child Adolesc Psychiatry. (2020) 29:11–27. 10.1007/s00787-019-01416-3 31845068

[50] Rouffet JB. Training Requirements for the Specialty of Child and Adolescent Psychiatry. Available online at: https://www.uems.eu/__data/assets/pdf_file/0019/44434/UEMS-2014.18-European-Training-Requirements-Child-adolescent-Psychiatry.pdf (accessed December 28, 2020).

[51] Gargot T Arnaoutoglou NA Costa T Sidorova O Liu-Thwaites N Moorey S. Can we really teach cognitive behavioral therapy with a massive open online course? Eur Psychiatry J Assoc Eur Psychiatr. (2020) 63:e38. 10.1192/j.eurpsy.2020.29 32151289 PMC7358632

[52] Revet A Hebebrand J Bhide S Caseiro J Conti E Deutz M. Dual training as clinician-scientist in child and adolescent psychiatry: are we there yet? Eur Child Adolesc Psychiatry. (2018) 27:263–5. 10.1007/s00787-017-1104-x 29318390

[53] Revet A Hebebrand J Klauser P. The 2017 ESCAP research academy workshop: bright perspectives for child and adolescent psychiatry. Eur Child Adolesc Psychiatry. (2017) 26:1279–80. 10.1007/s00787-017-1036-5 28808831

[54] Klauser P Hebebrand J Kehoe LA Huscsava M Revet A. The 2019 ESCAP research academy workshop: how novel technologies are impacting child and adolescent psychiatry. Eur Child Adolesc Psychiatry. (2020) 29:565–7. 10.1007/s00787-019-01378-6 31346738

[55] Verdoux H Tignol J. Focus on psychiatry in France. Br J Psychiatry J Ment Sci. (2003) 183:466–71. 10.1192/bjp.183.5.466 14649630

[56] Ravens-Sieberer U Erhart M Gosch A Wille N European KIDSCREEN Group. Mental health of children and adolescents in 12 European countries-results from the European KIDSCREEN study. Clin Psychol Psychother. (2008) 15:154–63. 10.1002/cpp.574 19115436

[57] Sjöström O Holst D. Validity of a questionnaire survey: response patterns in different subgroups and the effect of social desirability. Acta Odontol Scand. (2002) 60:136–40. 10.1080/000163502753740133 12166905

[58] Glick P. How reliable are surveys of client satisfaction with healthcare services? Evidence from matched facility and household data in Madagascar. Soc Sci Med. (2009) 68:368–79. 10.1016/j.socscimed.2008.09.053 19027216

